# Supplementary material for: Analysis of PPARγ Signaling Activity in Psoriasis
Source: Int J Mol Sci. 2021 Aug 10;22(16):8603. doi: 10.3390/ijms22168603 (PMC8395241; doi:10.3390/ijms22168603)
Supplement: Supplementary file 1 [file ijms-22-08603-s001.zip › Supplemental materials_Analysis of PPARg signaling activity in psoriasis/Pathway models/Models images and html files/PPARG inhibits phenotypes assotiated with psoriasis/244250.html]

hypercholesterolemia


# Disease hypercholesterolemia

|  |  |
| --- | --- |
| URN | urn:agi-meshdis:Hypercholesterolemia |
| Total Entities | 0 |
| Connectivity | 3427 |
| Name | hypercholesterolemia |

---

|  |  |
| --- | --- |
| Pathway | Protein Oxidation and Nitration Products as Disease Biomarkers |
|  | PPARG inhibits phenotypes assotiated with psoriasis |
|  | efects selected |
|  | New Combined Pathway |

---

|  |  |
| --- | --- |
| MedScan ID | 9010342 |

---

|  |  |
| --- | --- |
| Alias | hypercholesterinaemia |
|  | hyper-cholesterolemia |
|  | hypercholesterinemia |
|  | Hypercholesteremia |
|  | cholesteremia |
|  | cholesterinemia |
|  | cholesterolemias |
|  | excessive cholesterol in the blood |
|  | hyper-cholesterolemic |
|  | Hypercholerolemia |
|  | Hypercholesterolemias |
|  | Hypercholesteremias |
|  | Hypercholesterolemia |
|  | High Blood Cholesterol Level |
|  | Hypercholesteraemia |
|  | cholesterolemia |
|  | Elevated Cholesterol |
|  | hypercholesterolemic |

---

|  |  |
| --- | --- |
| MeSH ID | D006937 |
|  | M0010818 |

---

|  |  |
| --- | --- |
| MeSH Heading | Hypercholesterolemia |

---
